# Meta-Analysis: The Holy Grail?

**DOI:** 10.1007/s00270-026-04421-7

**Published:** 2026-04-06

**Authors:** Danielle J. W. Vos, Hannah H. Schulz, Martijn R. Meijerink

**Affiliations:** 1 Amsterdam UMC, University of Amsterdam, Amsterdam, The Netherlands; 2 Cancer Center Amsterdam, Amsterdam, The Netherlands

**Keywords:** IO: Interventional Oncology, EBM: Evidence-based medicine, MWA: Microwave ablation, RFA: Radiofrequency ablation, CA: Cryoablation, IRE: Irreversible electroporation, TARE: Transarterial radioembolization (TARE), TA(C)E: Trans arterial (chemo) embolization, RCTs: Randomized controlled trials, GRADE: Grading of recommendations assessment, development and evaluation, PRISMA: Preferred Reporting Items for Systematic Reviews and Meta-Analyses

## Abstract

**Introduction:**

Meta-analysis is often regarded as a cornerstone of evidence synthesis, yet its application in interventional oncology (IO) is challenged by heterogeneous study designs, rapidly evolving technologies, and limited availability of randomized controlled trials. This review evaluates the current state of evidence in IO, emphasizing the role of systematic reviews and meta-analyses in guiding clinical decision-making.

**Methods:**

To evaluate the level of evidence of the published studies within IO, a PubMed search was conducted with the aim to identify the various article types that have been published over the past decades.

**Results:**

Over the past decades, the number of original articles has remained relatively stable. A notable observation is the significant increase in the number of systematic reviews and meta-analyses, particularly when compared to the relatively unchanged volume of original articles.

**Discussion:**

This trend suggests a growing emphasis on evaluating existing research rather than generating new prospective studies. The grading of recommendations assessment, development and evaluation (GRADE) approach to assess evidence quality, the importance of adhering to Preferred Reporting Items for Systematic Reviews and Meta-Analyses (PRISMA) guidelines, and common challenges like risk of bias and publication bias will be discussed within this context.

**Conclusion:**

Ultimately, meta-analysis should not be viewed as a definitive benchmark in IO, but as a methodological tool of which conclusions depend heavily on the quality, consistency, and relevance of the underlying studies.

**Supplementary Information:**

The online version contains supplementary material available at 10.1007/s00270-026-04421-7.

## Introduction

Meta-analysis is often described as the ‘holy grail’ of evidence synthesis in clinical research, integrating the results of numerous clinical trials and observational studies, allowing researchers to identify treatment effects that might not be apparent in the individual studies [1]. This approach of utilizing the available data is particularly valuable in clinical research, where the heterogeneity of study populations and outcomes often makes it difficult to draw clear conclusions from single studies. In the field of interventional oncology (IO), where treatment options are diverse and treatment strategies are highly individualized, meta-analysis could play a crucial role in summarizing large datasets to guide clinical decision-making and change current guidelines [2, 3].

However, the question arises whether meta-analyses are always feasible in the relatively young and rapidly changing world of IO. Treatments in IO are frequently compared with those in more established disciplines, such as surgery, radiation oncology, and medical oncology, which either represent longstanding standards of care or are supported by numerous well-designed randomized controlled trials (RCTs) providing high levels of evidence. Though evidence would ideally come from well-conducted RCTs, in the field of IO, which is rapidly developing due to technological advancements and the increasing complexity of patients and (combination) treatments, retrospective small-scale comparisons play a significant role in evidence synthesis. Consequently, the quality of meta-analyses in IO is highly dependent on the methodological rigour of the included studies, raising questions about whether meta-analysis can consistently be regarded as the highest level of evidence in this field.

In this review, we discuss the Grading of Recommendations Assessment, Development, and Evaluation (GRADE) approach to evaluate the certainty of evidence in systematic reviews and meta-analyses and provide a snapshot of the current level of evidence of published articles within the field of IO. Additionally, the Preferred Reporting Items for Systematic Reviews and Meta-Analyses (PRISMA) guidelines are discussed to highlight their role in improving methodological quality and reporting transparency. We explore the importance of meta-analysis in clinical research in IO, highlighting its role in informing decision-making (e.g. guideline formulation), while addressing common challenges that may lead to misinterpretation or overestimation of treatment effects.

### What do we Publish in IO?

To evaluate the level of evidence of the published studies within IO, a PubMed search was conducted with the aim to identify the various article types that have been published over the past decades. Assessing IO as a whole proved to be challenging due to the diverse treatments at various stages of development, many of which are still being integrated into daily clinical practice and standard of care. Therefore, the results of this search should be interpreted with the understanding that the goal was to provide an overview of the current landscape of research in IO and assess the distribution of different study designs. The timeframe within the database was set from 2000 until 2025. The search strategy and corresponding terms are detailed in Appendix 1. In brief, the search query was structured using a primary term comprehending an IO treatment (e.g. ‘microwave ablation’) combined with an ‘AND’ operator to specifically include terms relevant to IO. This ensured that all publications contained at least one of the following oncology-related terms (e.g. ‘tumour’, ‘metastases’, ‘malignancy’, ‘carcinoma’, ‘lesion’, ‘cancer’, ‘neoplasm’). The search criteria were set to ensure that all resulting publications contained the search terms as well as at least one IO-related term in the title, abstract, or both. After conducting the search, the results were filtered by study type to refine the publications according to their methodological design. Subsequently, the number of published manuscripts for each study type was manually reviewed by examining PubMed article type (results by year section based on Medical Subject Headings (MeSH) and metadata from publishers), which displays the count of publications per article type for each year. This allowed for a rough analysis of the distribution of the following different study designs: (1) meta-analysis; (2) systematic review; (3) phase 3 and 4 trials including RCTs; and (4) phase 1 and 2 trials.

The study by Kaufmann et al. employed a similar search strategy to evaluate the level of evidence in interventional oncology (IO) based on articles published between 2012 and 2022 [4]. However, their approach included more detailed analysis of the varying types of published articles. Their findings indicated that RCTs accounted for only 7% of the literature, while non-randomized clinical studies were more common, comprising approximately 20%. The majority of publications in IO were case reports (68%). Notably, Kaufmann et al. did not research and report on the amount of systematic reviews and meta-analysis that have been published in relation to the other study types.

**Fig. 1:**
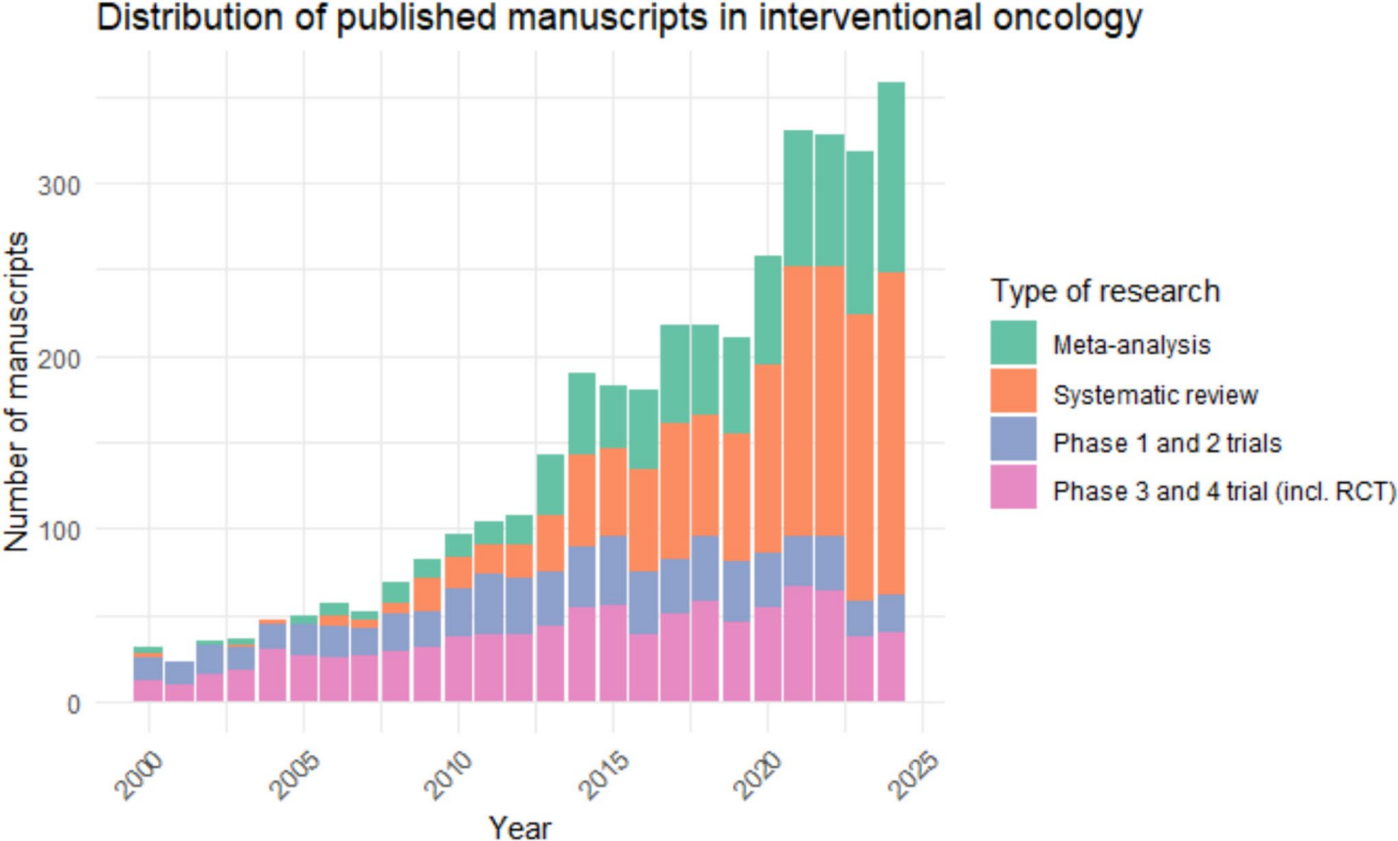
Distribution of published manuscripts on Pubmed in interventional oncology from 2000–2024

The results of the search conducted in this review are presented in Fig. 1. Over the past decades, the number of original articles has remained relatively stable. Consequently, it is reasonable to assume that the distribution of the different study types within these original articles has remained similar as presented by Kaufmann et al. A notable observation is the significant increase in the number of systematic reviews and meta-analyses, particularly when compared to the relatively unchanged volume of original articles. This trend suggests a growing emphasis on evaluating existing research rather than generating new prospective studies.

### Preferred Reporting Items for Systematic Reviews and Meta-Analyses (PRISMA)

**Fig. 2:**
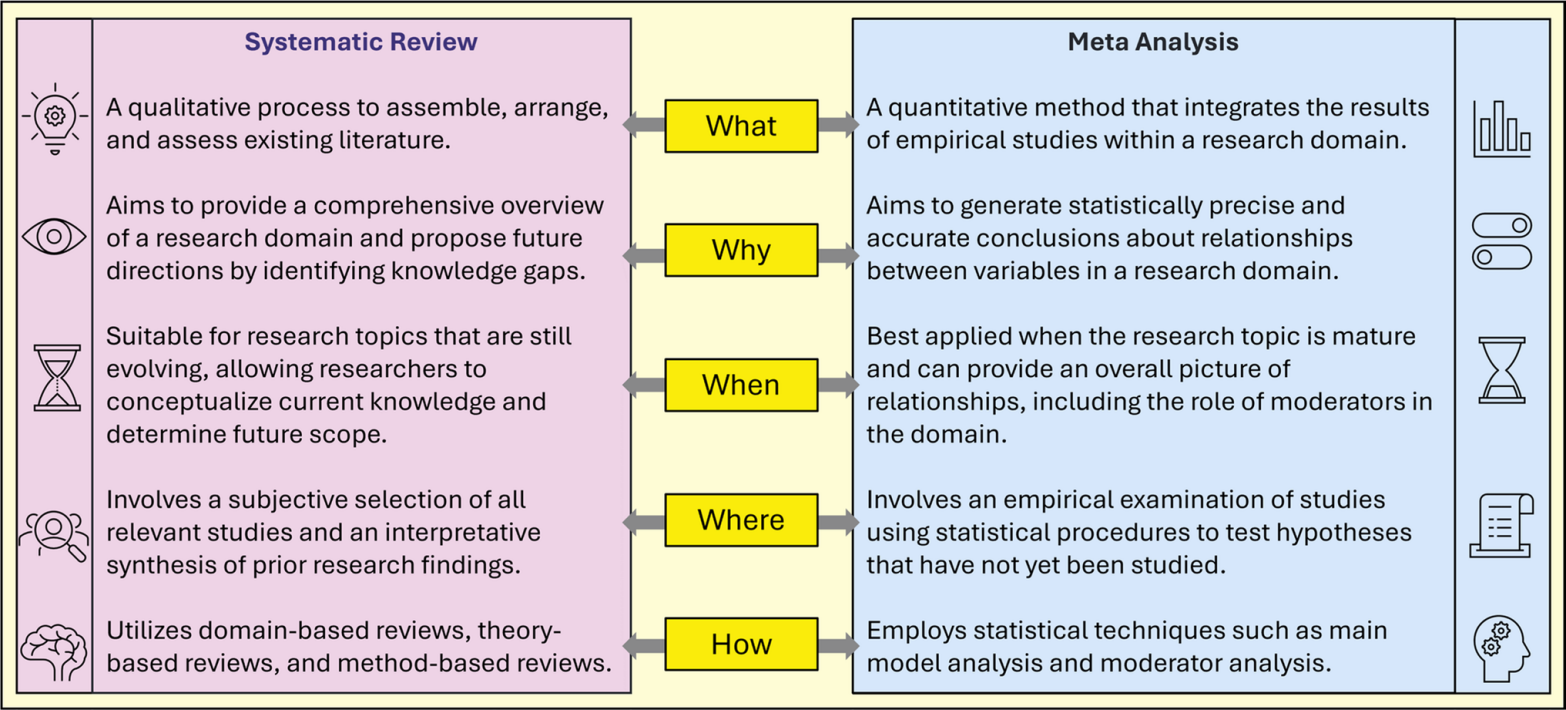
Systematic reviews and meta-analyses are closely related methods that complement each other in evidence synthesis. While a meta-analysis focuses on statistically combining data from multiple studies to derive a comprehensive result, it relies on the structured approach of a systemic literature review to identify and evaluate relevant research. To conduct a high-quality meta-analysis, understanding the key steps in a systematic review is essential

As a standard practice, both systematic reviews and meta-analyses should be conducted and reported in accordance with the PRISMA (Preferred Reporting Items for Systematic Reviews and Meta-Analyses) guidelines, which aim to improve the transparency, consistency, and completeness of systematic reviews and meta-analyses [5]. Following these guidelines enhances the reliability of findings, increases statistical power, and reduces the risks associated with small sample sizes and biased study designs. While both methodologies synthesize findings from multiple studies, a key distinction lies within the approach to data analysis (Fig. 2). A systematic review critically appraises and summarizes existing research following a structured and reproducible methodology but does not statistically combine the quantitative data from individual studies. In contrast, a meta-analysis goes a step further by pooling and analysing data quantitatively, thereby increasing statistical power and providing a more precise estimate of the true effect of an intervention. This positions meta-analyses the highest attainable level of evidence, as it not only mitigates the limitations of individual studies but also offers a more robust and generalized understanding of treatment effects, particularly when individual trials yield conflicting results. Nevertheless, it is important to acknowledge that systematic reviews and meta-analyses are susceptible to several limitations, such as bias, when non-randomized studies are included, and the quality of evidence should be evaluated when interpreting meta-analyses.

### Quality of Evidence and Strength of Recommendations (GRADE)

**Fig. 3:**
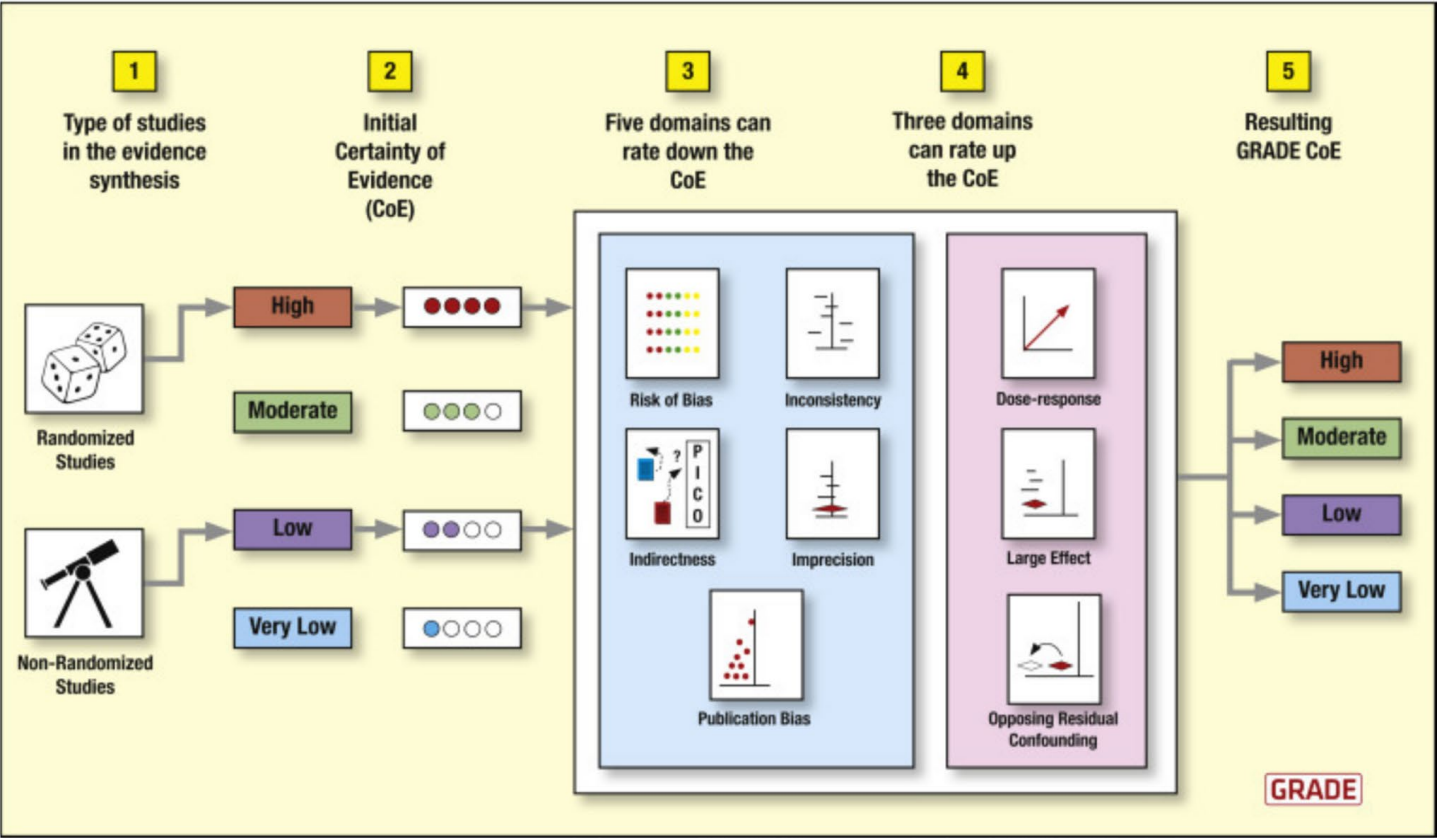
Grading of Recommendations, Assessment, Development, and Evaluation (GRADE) rating quality of evidence in systematic reviews and guidelines of interventions. In the GRADE approach, randomized trials start as high-quality evidence and observational studies as low-quality evidence, but both can be rated down if there are serious concerns in any GRADE domains and rated up if specific criteria are met. Adapted from GRADE handbook and based on GRADE meeting, Edinburgh 2009. (https://gdt.gradepro.org/app/handbook/handbook.html). Image redrawn by Becky Drager of Drager Studios from original art by Dr Carlos Cuello-Garcia (McMaster University, GRADE WG member, @CharlieNeck)

The Grading of Recommendations Assessment, Development, and Evaluation (GRADE) framework is currently the most widely accepted approach for assessing the quality of evidence and strength of recommendations in clinical research. It provides a systematic method for evaluating how confidently evidence can inform clinical decision-making. GRADE classifies evidence into four levels—high, moderate, low, and very low—reflecting the degree of certainty in the reported findings [6]. Figure 3 represents an overview of the GRADE framework.

Within the GRADE framework, the initial certainty of evidence is determined by study design. Evidence derived from randomized controlled RCTs starts as high-quality evidence, whereas evidence from non-randomized or observational studies starts as low-quality evidence. This initial rating is not fixed but can subsequently be downgraded or upgraded based on predefined criteria [7].

Evidence may be downgraded if there are serious concerns in one or more of five domains: risk of bias, inconsistency, indirectness, imprecision, or publication bias [7]. These domains capture common methodological limitations that may reduce confidence in the observed effect estimates, regardless of statistical significance. In contrast, observational studies may be upgraded if they demonstrate a large magnitude of effect, a clear dose–response relationship, or if all plausible residual confounding would be expected to reduce the observed effect rather than exaggerate it [8]. These upgrading criteria acknowledge that, under certain conditions, non-randomized evidence can provide a high degree of confidence in treatment effects, as illustrated in Fig. 3. The GRADE framework shows that statistical significance alone is insufficient to determine the certainty of evidence. P values merely reflect the probability of observing the data under the null hypothesis and do not convey information on effect size, clinical relevance, or methodological quality. Consequently, statistically significant results may still be downgraded within the GRADE framework if they arise from studies with limited internal validity, lack of randomization, or substantial risk of bias, while non-significant findings may still contribute valuable evidence when supported by adequate study design and precision. This distinction underscores the importance of evaluating statistical results in the context of overall study quality rather than relying on P values as a proxy for evidence certainty.

By incorporating these principles, the GRADE system enhances transparency in evidence evaluation and ensures that clinical recommendations are based on both the quality of available data and its clinical applicability. This structured approach aids healthcare professionals in making well-informed decisions, ultimately improving patient care. In the following sections, examples from interventional oncology will illustrate how different GRADE domains may influence the certainty of evidence.

## Evidence Downgrading

### Risk of Bias

All RCTs and observational studies have a risk of bias, or in other words, a risk of a systemic error in studies that leads to inaccurate deductions or conclusions [9]. For IO studies in particular, there is a pronounced risk of residual confounding. Residual confounding is defined as confounding that persists even after statistical adjustment for known and measured variables. As a result, true causal relationships between treatment and outcome can be difficult to establish. Residual confounding may therefore partly explain why meta-analyses and large RCTs investigating the same intervention can yield divergent results. This phenomenon was illustrated in the review by LeLorier et al. [10], which demonstrated that previously published meta-analyses failed to accurately predict the outcomes of subsequent large RCTs in 35% of cases across 12 clinical topics. An illustrative example within IO is the COLLISION trial, which compared local treatment strategies for colorectal liver metastases (CRLM), namely surgical resection versus thermal ablation. The RCT demonstrated a high likelihood of non-inferiority of thermal ablation with respect to OS and local tumour control in patients with small (< 3 cm) CRLM [11]. In contrast, several meta-analyses conducted prior to the initiation of the COLLISION trial concluded that thermal ablation was inferior to surgical resection in terms of overall survival [12, 13].

These discrepancies can plausibly be attributed to residual confounding in the observational studies included in the meta-analyses. Specifically, patients selected for surgical resection were often compared with patients in whom surgery was not feasible or no longer considered an option due to unfavourable patient- or disease-related characteristics. Such systematic differences between treatment groups are difficult to fully adjust for and may have biased the meta-analytic results against thermal ablation. This example of residual confounding underscores the limitations of observational evidence and highlights the continued need for well-designed prospective studies, particularly RCTs, in IO.

To assess the risk of bias, authors can use standardized, design specific tools such as the Cochrane Risk of Bias 2 (RoB 2) tool for RCTs and the Risk Of Bias In Non-randomized Studies of Interventions (ROBINS-I) tool for observational studies. This can be used as part of GRADE’s certainty rating process, assigning colour-coded scores, as shown in Fig. 3: green (low risk), yellow (some concerns), and red (high risk).

RoB 2 evaluates bias across five domains that may occur despite randomization, including the randomization process itself, deviations from intended interventions, missing outcome data, outcome measurement, and selective reporting. In contrast, ROBINS-I assesses seven domains and is specifically designed to account for biases inherent to non-randomized designs, such as confounding and selection bias, by comparing the study to a hypothetical, well-conducted target trial. As a result, ROBINS-I generally applies more stringent criteria, and non-randomized studies are more likely to be rated at higher risk of bias than RCTs [14].

These scores are visualized in traffic light plots or bar charts to help interpret bias across studies and allow for sensitivity analyses to assess the impact of bias on overall findings [15]. In case of a high risk of bias, the evidence may need to be downgraded.

### Serious Indirectness

Serious indirectness occurs when study findings are not directly applicable to the target population, intervention, comparator, or outcome [16]. An example of evidence downgrading because of serious indirectness is the meta-analysis by Meijerink et al., published in CVIR, which assessed the efficacy of radiofrequency ablation (RFA) for colorectal liver metastases (CRLM) compared to chemotherapy alone [12]. The authors had to downgrade the evidence level due to serious indirectness, as the randomized controlled CLOCC trial included patients who also underwent surgical resection alongside RFA, creating a double comparator. Furthermore, the trial was stopped early due to slow accrual, failing to reach its pre-planned sample size, which led to serious imprecision due to broad confidence intervals. This premature publication contributed to an underestimation of the true long-term benefits in the initially negative results. However, the later-published long-term follow-up demonstrated a significant survival benefit, ultimately leading to the global adoption of ablation as the standard of care for unresectable CRLM [17]. Another example of serious indirectness that occurs in the field of IO is the geographical concentration of studies included in meta-analyses, such as in the treatment of hepatocellular carcinoma (HCC). Several meta-analyses evaluating locoregional therapies, such as RFA or MWA and Transarterial radioembolization (TARE), predominantly include studies from Asian or Western countries [18, 19]. While these data reflect regions with a high incidence of HCC, their applicability to Western or Asian populations may be limited. Differences in underlying liver disease aetiology (predominantly hepatitis B–related cirrhosis in Asia versus metabolic- or alcohol-associated liver disease in Western countries [20], tumour stage at diagnosis, surveillance intensity, patient selection criteria, and procedural expertise may plausibly modify treatment effects. According to the GRADE framework, such population and setting differences constitute serious indirectness when extrapolating results to certain regional clinical practice, and may justify downgrading the certainty of evidence [16].

### Publication Bias

As in many other medical research fields, publication bias represents a significant methodological concern in meta-analyses within IO [21, 22]. Studies reporting statistically significant or favourable outcomes are more likely to be published, whereas studies with negative or inconclusive results may remain unpublished. This selective dissemination can distort the available evidence base and lead to an overestimation of treatment efficacy in meta-analytic conclusions [23]. An illustrative example is the SARAH trial, a well-conducted open-label randomized controlled phase 3 study comparing TARE with sorafenib in patients with locally advanced and inoperable HCC [24]. This trial demonstrated a median OS of 8 months in the TARE group versus 9.9 months in the sorafenib group. Notably, the sample size calculation for this trial was based on a literature review reporting a pooled median OS of 15 months (range 7–26.7 months) for TARE [24]. The discrepancy between the OS estimated from predominantly retrospective studies and the OS observed in this RCT suggests that the combined retrospective series may have overestimated treatment effects. One plausible explanation is publication bias.

Another intriguing example of possible publication bias is discussed in the scoping review of oncology meta-analyses of Haslam et al. This study showed that industry payments related to the intervention or study sponsorship were linked to a higher likelihood of a meta-analysis reporting favourable results for an intervention, compared to non–industry-funded studies [25]. Since IO is a specialty that closely collaborates with the industry, this outcome may also apply to studies within IO.

As such, authors of meta-analyses should be aware of the risk of publication bias when available evidence comes from a number of small studies and when most of which have been commercially funded. Authors can assess publication bias by using both graphical, such as Funnel plots, and statistical method, such as the Egger’s test [22]. Funnel plots display individual study effect estimates against study precision, in which the larger, more precise studies cluster near the top of the plot and tend to lie close to the pooled effect estimate, whereas smaller studies appear lower in the plot and show greater scatter. In the absence of publication bias, this scatter is expected to be symmetrical around the either the point estimate (dominated by the larger trials) or the results of the larger trials themselves, forming an inverted funnel. Asymmetry arises when smaller studies are not evenly distributed around the pooled effect, for example when small studies with negative or non-significant results are missing, leading to a skewed funnel. Egger’s regression test provides a quantitative assessment of funnel plot asymmetry by testing whether smaller, less precise studies tend to report systematically different effects than larger, more precise studies [26]. However, both funnel plots and Egger’s test have important limitations, particularly in meta-analyses with few studies or substantial heterogeneity, conditions common in IO research. Therefore, indications of asymmetry should be interpreted cautiously and in conjunction with qualitative assessment of study design, methodological quality, and clinical heterogeneity. However most importantly, well-designed RCTs and preregistration of clinical trials and meta-analysis protocols help reduce the risk of publication bias [27].

### Evidence Upgrading

Conversely, observational studies can be upgraded if they demonstrate a large effect size, a dose–response gradient, or if plausible confounding would likely underestimate the true effect [8]. As a well-known example the Barcelona Clinic Liver Cancer (BCLC) upgraded the evidence level supporting trans-arterial chemoembolization (TACE) for intermediate-stage HCC based on two small-size RCTS and a multitude of observational studies demonstrating a large effect size, dose–response gradient with repeated treatments, and plausible confounding that likely underestimated its true survival benefit, ultimately leading to its adoption as the standard of care in major clinical guidelines [28].

## Specific Challenges for Meta-Analysis in IO

Multiple challenges hinder the suitability of IO research for systematic reviews and meta-analyses of which three will be discussed in this review lack of standardization, rapid technological advancement and varying levels of experience among centres and operators.

Variability in methodologies, endpoints, and outcome measures can lead to significant heterogeneity and can limit the interpretability and clinical applicability of IO research This is exemplified by the systematic review by Spiers et al. on the efficacy of irreversible electroporation (IRE) for CRLM, in which meta-analysis was not feasible due to the heterogenous, as well as inconsistent efficacy data and unclear aetiology in the included studies [29]. One of the cause of this found heterogeneous data is the large variety of oncological outcomes used in IO [30]. Even though OS remains the only fully validated time-to-event endpoint, there is a need for surrogate endpoints, such as PFS, to reduce trial duration, costs and required sample sizes [31]. However, these surrogate endpoints are frequently inconsistently or poorly defined, with definitions varying across trials, thereby limiting comparability and potentially influencing estimates of treatment effect. For future studies and meta-analyses, data and oncology endpoints need to be more standardized and there should be higher-quality reporting. To address this issue, a modified Delphi consensus project proposed standardized definitions and reporting recommendations for patient-, session-, and tumour-level oncologic outcomes [32]. While this framework represents an important step towards harmonization, further research is needed to determine which time-to-event endpoints are the most valid surrogates for OS in the overall IO setting [30].

Secondly, IO is a rapidly evolving field characterized by continuous technological advances in devices, imaging guidance, and procedural techniques. Results from older studies may not accurately reflect current practices or the impact of new technologies. A meta-analysis that includes studies spanning several years or decades may struggle to account for these changes, leading to a possible dilution or misinterpretation of the true effect of an intervention. IO has to compete and collaborate with more established specialties, and progressive insight and technological advancements may still need to be recognized by both ourselves and our colleagues. An illustrative example is the meta-analysis by Buisman et al. in which the surgical placement of the intrahepatic pump is compared to the percutaneous placement to allow continuous regional chemotherapy delivery directly into the hepatic artery. There are only two small studies on percutaneous placement compared to 13 surgical studies [33]. The authors do not make a direct comparison but state that the percutaneous approach has been abandoned due to high complication rates and dislodgement. However, the authors refer to studies from 1977 to 2000. It is expected, however, that substantial technical advancements have been made in percutaneous catheter implantation in the past 25 years.

When conducting meta-analyses, authors should consider stratifying analyses by era of technology or device generation to account for temporal improvements in technique and equipment. This can help clarify whether observed effects reflect true treatment efficacy or historical practice patterns.

Thirdly, as IO is a rapidly evolving field, the variable experience among centres and operators performing IO procedures should also be taken into account when conducting meta-analysis. Volume of procedures has a proven positive relationship to procedure outcome in the surgical field [34] and is probably the same for IO. Additionally, IO techniques often entail a steep learning curve, such as is the case with RFA for liver tumours. A study by Poon et al. showed that complication rates and complete ablation rates improved with experience [35]. These experience- and volume-related differences complicate the design of RCTs and consequently the interpretation of meta-analyses in IO.

It emphasizes the need for formalized training, credentialing standards and consensus guidelines, as this could help reduce variability in operator performance and improve the consistency of clinical outcomes. An example of such a consensus guideline is the Ruarus consensus for the relative standardization of hepatic IRE, which provides expert-based recommendations on the technical and clinical aspects of hepatic IRE, including patient selection, imaging guidance, probe placement, energy delivery parameters, and peri-procedural management.[29, 36]. Given the impact of experience on procedural success, such frameworks could be required for participation in multicentre clinical trials. Additionally, authors could report detailed information on operator experience and centre volumes. Such transparency can enable subgroup analyses or meta-regression approaches that explore the influence of experience on outcomes.

## Conclusion

In conclusion, meta-analysis remains a powerful tool for synthesizing evidence in IO. However, its potential can be compromised by issues such as publication bias, residual confounding, and serious indirectness, all of which must be carefully considered when interpreting results. The heterogeneity of treatment strategies, patient populations, and study designs presents significant challenges to the reliability and generalisability of pooled estimates. Moreover, the rapid pace of technological advancements in IO introduces an additional layer of complexity, as older studies may no longer reflect current clinical practice. Moreover, variability in operator experience and centre volume should be acknowledged. Greater standardization of protocols and the development of consensus guidelines are essential to strengthen the evidence base. Ultimately, the quality of meta-analytic evidence remains highly dependent on the methodological rigour of the included studies.

In this context, meta-analysis cannot be regarded as a ‘holy grail’ in the field of IO, but rather as a valuable methodological tool whose conclusions are inherently dependent on the quality, relevance, and consistency of the underlying evidence. While meta-analyses can meaningfully inform clinical decision-making when conducted and interpreted with appropriate methodological caution, they may also convey a false sense of certainty if their limitations are insufficiently acknowledged.

As IO continues to mature, rigorous study design and adherence to evidence evaluation frameworks such as GRADE, combined with transparent and structured reporting in accordance with PRISMA, will be crucial to enhance the reliability and clinical applicability of meta-analytic findings.

## Supplementary Information

Below is the link to the electronic supplementary material.

Supplementary file1 (DOCX 15 kb)
