# Supplementary material for: Meta-Analysis: The Holy Grail?
Source: Cardiovasc Intervent Radiol. 2026 Apr 6;49(5):883–91. doi: 10.1007/s00270-026-04421-7 (PMC13156232; doi:10.1007/s00270-026-04421-7)
Supplement: Supplementary file 1 — Supplementary file1 (DOCX 15 kb) [file 270_2026_4421_MOESM1_ESM.docx]

| **Primary search term comprehending an IO treatment** |  | **Search terms relevant to IO** |
| --- | --- | --- |
| ("radiofrequency ablation"[tiab]) OR  ("microwave ablation"[tiab]) OR  ("embolization"[tiab] OR  "embolisation"[tiab] OR  "bland embolization"[tiab] OR  bland embolisation[tiab] OR  "transarterial embolization"[tiab] OR  "transarterial embolisation"[tiab]) OR  ("cryoablation"[tiab]) OR  ("TACE"[tiab] OR  "DEB-TACE"[tiab] OR  "DSM-TACE"[tiab] OR  "DEM-TACE"[tiab] OR  "STARCH microspheres"[tiab] OR  "microspheres"[tiab] OR  "beads"[tiab] OR  "chemoembolization"[tiab] OR  "chemoembolisation"[tiab] OR  "chemosaturation"[tiab]) OR  ("High-intensity focused ultrasound"[tiab] OR  "High intensity focused ultrasound"[tiab]) OR  ("Laser ablation"[tiab] OR  "Laser-induced thermotherapy"[tiab]) OR  ("Electrochemotherapy"[tiab] OR  "Irreversible electroporation"[tiab]) OR  ("high dose rate interstitial brachytherapy"[tiab] OR  "high-dose rate interstitial brachytherapy"[tiab] OR  hdr interst. bt[tiab] OR  "hdr brachytherapy"[tiab]) OR  ("percutaneous ethanol injection"[tiab]) OR  ("intra-arterial chemotherapy"[tiab] OR  "intraarterial chemotherapy"[tiab] OR  "hepatic arterial infusion"[tiab] OR  "transarterial chemotherapy"[tiab]) OR  ("radioembolization"[tiab] OR  "radioembolisation"[tiab] OR  "sirt"[tiab] OR  "selective internal radiation therapy"[tiab]) | **AND** | ("tumor"[tiab] OR  "tumour"[tiab] OR  "metastases"[tiab] OR  "metastasis"[tiab] OR  "malignant"[tiab] OR  "malignancy"[tiab] OR  "carcinoma"[tiab] OR  "lesion"[tiab] OR  "cancer"[tiab] OR  "neoplasm"[tiab] OR  "sarcoma"[tiab] OR  "stromatumour"[tiab] OR  "stroma tumour"[tiab] OR  "stromaltumour"[tiab] OR  "stromal tumour"[tiab] OR  "gastrointestinal"[tiab] OR  "gastro intestinal"[tiab]) |

**Appendix 1.** Supplementary Table: Pubmed search strategy; the search query was structured using a primary term comprehending an IO treatment combined with an ‘AND’ operator to specifically include terms relevant to IO (oncology-related terms). The search criteria were set to ensure that all resulting publications contained the search terms as well as at least one IO-related term in the title, abstract, or both. After conducting the search, the results were filtered by study type to refine the publications according to their methodological design. Subsequently, the number of published manuscripts for each study type was manually reviewed by examining the left-hand column in PubMed (results by year section), which displays the count of publications per article type for each year. This allowed for a rough analysis of the distribution of the following different study designs: (1) meta-analysis; (2) systematic review; (3) phase 3 and 4 trials including randomized controlled trials (RCTs); and (4) phase 1 and 2 trials.
